# Modeling acute toxicity of metal mixtures to wheat (*Triticum aestivum* L.) using the biotic ligand model-based toxic units method

**DOI:** 10.1038/s41598-017-09940-5

**Published:** 2017-08-25

**Authors:** Mingyan Wu, Xuedong Wang, Zhiguo Jia, Karel De Schamphelaere, Dongxue Ji, Xiaoxiu Li, Xiaolin Chen

**Affiliations:** 1 The Key Lab of Resource Environment and GIS, College of Resource Environment and Tourism, Capital Normal University, Beijing, 100048 P. R. China; 2 Department of Horticulture, Hebei North University, Zhangjiakou, 075000 P. R. China; 3 Laboratory of Environmental Toxicology and Aquatic Ecology, Environmental Toxicology Unit, Ghent University, Ghent, B-9000 Belgium

## Abstract

The combined toxic effects of copper (Cu) and cobalt (Co) were predicted using the biotic ligand model (BLM) for different concentrations of magnesium (Mg^2+^) and pH levels, with parameters derived from Cu-only and Co-only toxicity data. The BLM-based toxic unit (TU) approach was used for prediction. Higher activities of Mg^2+^ linearly increased the EC_50_ of Cu and Co, supporting the concept of competitive binding of Mg^2+^ and metal ions in toxic action. The effects of pH on Cu and Co toxicity were related not only to free Cu^2+^ and Co^2+^ activity, respectively, but also to inorganic metal complexes. Stability constants for the binding of Cu^2+^, CuHCO_3_^+^, CuCO_3_(aq), CuOH^+^, Mg^2+^, Co^2+^, CoHCO_3_^+^ and Mg^2+^ with biotic ligands were log*K*_CuBL_ 5.87, $\mathrm{log}\,{K}_{{{\rm{CuHCO}}}_{3}{\rm{BL}}}$ 5.67, $\mathrm{log}\,{K}_{{{\rm{CuCO}}}_{3}{\rm{BL}}}$ 5.44, log*K*_CuOHBL_ 5.07, log*K*_MgBL_ 2.93, log*K*_CoBL_ 4.72, $\mathrm{log}\,{K}_{{{\rm{CoHCO}}}_{3}{\rm{BL}}}$ 5.81 and log*K*_MgBL_ 3.84, respectively. The combinations of Cu and Co showed additive effects under different conditions. When compared with the FIAM-based TU model (root mean square error [*RMSE* = 16.31, *R* ^2^ = 0.84]), the BLM-based TU model fitted the observed effects better (*RMSE* = 6.70, *R* ^2^ = 0.97). The present study supports the BLM principles, which indicate that metal speciation and major cations competition need to be accounted for when predicting toxicity of both single metals and mixtures of metals.

## Introduction

With the acceleration of industrialization and the agriculture industry, large amounts of metals have been released into the soil, where they can pose risks to the environment^1^. For example, environmental copper (Cu) concentrations are frequently elevated because of sewage sludge and animal manure, burning of fossil fuels, industrial processes and widespread use of pesticides^2^. Nationwide surveys in China recently reported that 19% of agricultural soils are polluted, mainly with heavy metals and metalloids—among which 2.1% of samples exceeded China’s soil environmental quality for Cu^3^. The minerals cobaltite, smaltite and erythrite contain cobalt (Co), and anthropogenic activities such as mining and smelting can lead to Co contamination of soil in some areas^4^. Soil contamination is complicated, and organisms in the soil are often simultaneously exposed to multiple metal elements^5^. However, risk-evaluation is usually based on the effects of individual metals in soils^6^. Therefore, investigation and assessment of the environmental effects on mixtures of metals such as Cu–Co may have practical significance.

The concentrations of individual toxicants in a mixture cannot be assessed based simply on their added concentrations. One of the models most commonly used to assess the toxicity of chemical mixtures is the toxic unit (TU) approach^5^, which is employed to calculate the sum of the concentrations of individual chemicals divided by their median effective concentrations (EC_50_)^7^.

1 $${\rm{TU}}=\sum {{\rm{TU}}}_{i}=\sum _{i=1}^{n}\frac{{{\rm{M}}}_{{\rm{i}}}^{2+}}{{{\rm{EC50}}}_{i}}$$

where, *i* is the identity of the metal, *n* is the number of metals, ${{\rm{M}}}_{{\rm{i}}}^{2+}$ is the activity of free metal ions such as Cu^2+^ and Co^2+^, and EC50_i_ is the median effective concentration of a single metal. The TU model is commonly used to normalize the toxic impacts and categorise the type of combined effect of metal mixtures. In multi-metal systems, a metal mixture is termed additive when TU is equal to 1 at 50% effect, while it is called synergistic or antagonistic when TU is less than or greater than 1^8^. The TU approach has been successfully used to predict joint toxicity and as a tool to assess the corresponding combined effects. However, conventional studies using the TU approach to examine the combined toxicity of multiple metals do not consider the speciation and phytotoxicity of metals under different environmental conditions.

Recently, the biotic ligand model (BLM) has become popular for evaluating metal bioavailability and toxicity in aquatic and terrestrial systems^9^. The critical assumption of the BLM is that metal toxicity depends on free metal ions (or other reactive metal species), which can react with biological binding sites and form a metal–biotic ligand (BL) complex. This model assumes that the cations (e.g., K^+^, Na^+^, Mg^2+^, Ca^2+^) compete with metals for binding sites to mitigate the toxicity of free metal ions^10^. Generally, BLMs are considered to be useful tools for evaluating the toxic effects of metals on organisms. Most previous studies using the BLM to evaluate the impacts of metals have been applied to individual metals. However, it is also increasingly being applied to the assessment of the effects of mixtures of metals. Jho *et al*. found that data describing the toxicity of a single metal can be used in the BLM-based TU method to predict the joint toxicity of Cd–Pb mixtures to *Vibrio fischeri* ^1^. Although terrestrial higher plants also appear suitable for these types of studies, they have rarely been used in investigations of alternative methods for joint toxicity of metals. To the best of our knowledge, only three reports have employed the BLM to estimate the combined toxicity of metals on joint toxicity^10–12^.

When developing the BLM for individual metals, some studies indicated that the toxicity of metals to plants was partly dependent on inorganic complexes (e.g., carbonate and hydroxide species) when exposed to relatively high pH^13^. However, no published studies of combined toxicity of mixtures of metals have considered the potential effects of these metal complexes^12, 14, 15^. Furthermore, most BLMs for metal mixtures have considered Ca^2+^ competition, while ignoring Mg^2+^ competition^1, 10^. Earlier studies of BLM for single metals showed that Mg^2+^ has a greater impact on the toxicity of some single metals (e.g., Co and Cu) than Ca^2+^ ^16, 17^. Therefore, the present study was conducted to investigate the joint toxicity of Cu–Co mixtures toward wheat (*Triticum aestivum* L.) in the presence of different Mg^2+^ concentrations and under a wide range of pH values using the BLM-based TU method^1^. In addition, the traditional free ion activity model (FIAM)-based TU approach^18^ was employed for combined toxicity prediction and the results were compared with those obtained using the BLM-based TU method.

## Results

### Toxicity of Cu^2+^ and Co^2+^ individually

**Figure 1:**
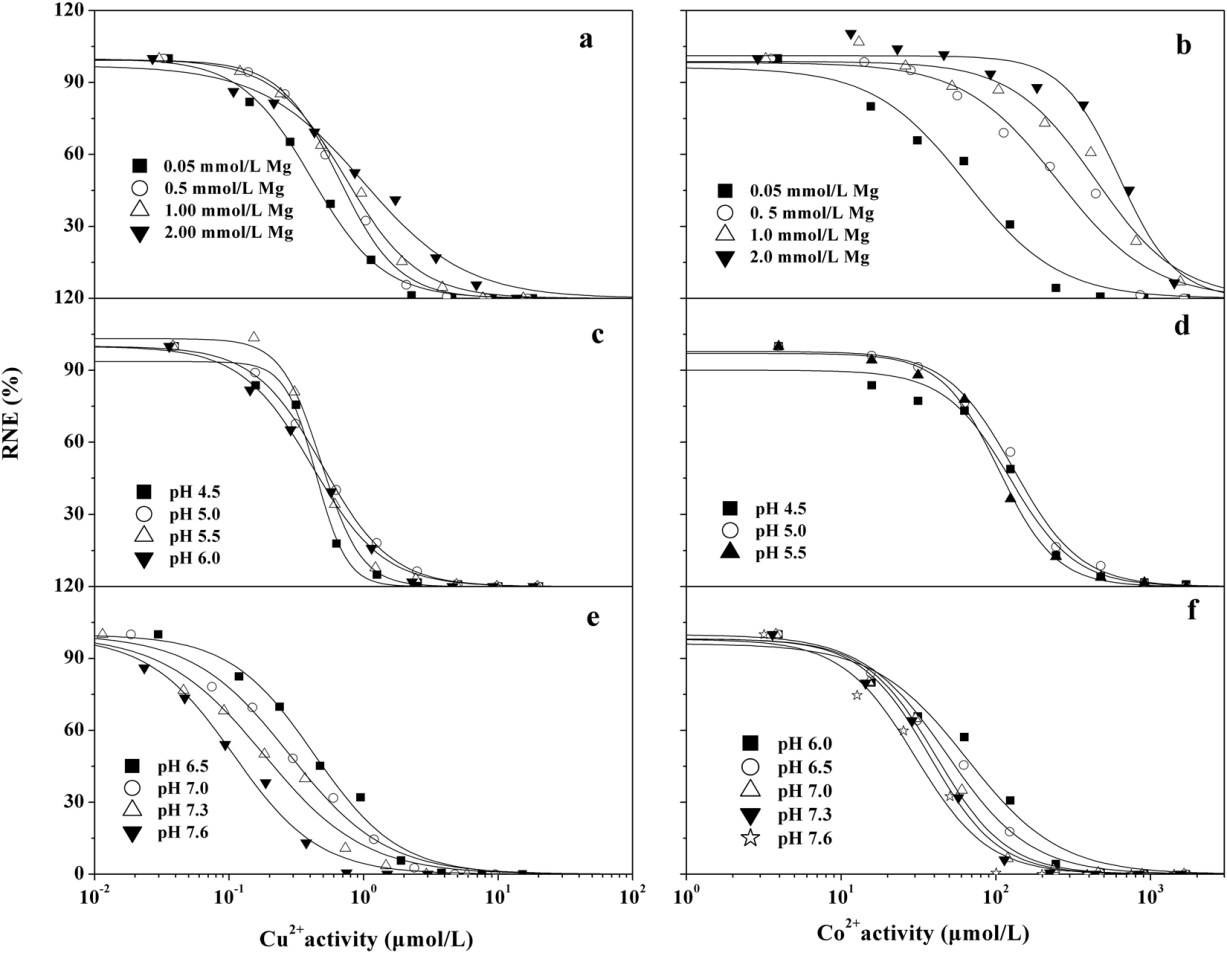
Dose–response relationships between relative net elongation (RNE, %) of wheat and free metal ion activity: free Cu^2+^ activity (first column) and free Co^2+^ activity (second column) under different Mg treatments (first row) and pH levels (second row for Cu^2+^, third row for Co^2+^). Each series point represents the observed RNE at the corresponding solution of Mg and pH treatment. The solid lines are fitted using log-logistic curves (RNE = 100/{1 + [M^2+^ */*EC50{M^2+^}]^*β*^_M_}). EC_50_ values and the slopes estimated based on these log-logistic curves are reported in Table 1.

A summary of the median effective concentration (EC_50_) for wheat root elongation expressed as free Cu^2+^ and Co^2+^ activity at different Mg^2+^ concentrations and pH level is given in Table 1. Dose–response curves for both free Cu^2+^ and Co^2+^ activities were established in Fig. 1. The increasing Mg^2+^ activities resulted in corresponding reductions in free Cu^2+^ activity by 2.5-fold and free Co^2+^ activity by 10-fold, respectively (Table 1). Furthermore, a linear relationship was found between Mg^2+^ activities and EC_50_ of Cu^2+^ or Co^2+^ activities (Cu^2+^: *p < *0.01, *R* ^2^ = 0.97; Co^2+^: *p* < 0.01, *R* ^2^ = 0.97). These results suggest that Mg^2+^ can compete with Cu^2+^ and Co^2+^ for binding sites on wheat root and that it can alleviate the toxicity of Cu and Co. Previous studies showed that K^+^ and Na^+^ did not significantly influence the toxicity of Cu and Co to wheat and barley, and that the effects of Ca^2+^ on the toxicity of Cu^2+^ and Co^2+^ were only slightly larger than K^+^ and Na^+^, and smaller than the effects of Mg^2+^ ^13, 17, 19^. Therefore, the effects of K^+^, Na^+^ and Ca^2+^ were ignored and not incorporated into the BLM in the present study.

**Table 1:** Composition of the test media, and the fitted individual EC_50_ (expressed as free ion activity) of Cu-only, Co-only and TU_50_ of Cu–Co mixture in the various bioassay sets using the log-logistic relationship.

Bioassay set		Characteristics of background solutions	EC50(µM)		EC50(µM)		TU50	*β*
			Cu-only	*β*	Co-only	*β*	Cu + Co	
Mg	0.05 mM	0.08 K, 2.0 Na, 0.2 Ca (mM), pH 6.0	0.41	3.76	64.49	3.31	1.03	4.89
Mg	0.5 mM	0.08 K, 2.0 Na, 0.2 Ca (mM), pH 6.0	0.66	4.5	251.52	3.15	0.93	5.77
Mg	1.0 mM	0.08 K, 2.0 Na, 0.2 Ca (mM), pH 6.0	0.74	3.71	438.34	3.73	1.11	3.25
Mg	2.0 mM	0.08 K, 2.0 Na, 0.2 Ca (mM), pH 6.0	1.01	2.63	620.75	5.5	0.97	5.12
pH	4.5	0.08 K, 2.0 Na, 0.2 Ca, 0.05 Mg (mM)	0.45	8.73	122.6	4.74	0.88	5.21
pH	5	0.08 K, 2.0 Na, 0.2 Ca, 0.05 Mg (mM)	0.5	3.93	129.42	4.63	1.1	6.49
pH	5.5	0.08 K, 2.0 Na, 0.2 Ca, 0.05 Mg (mM)	0.49	6.71	104.01	5.4	1.15	8.26
pH	6	0.08 K, 2.0 Na, 0.2 Ca, 0.05 Mg (mM)	0.42	3.76	64.61	3.35	1.02	4.89
pH	6.5	0.08 K, 2.0 Na, 0.2 Ca, 0.05 Mg (mM)	0.43	3.2	55.11	3.77	1.04	6.57
pH	7	0.08 K, 2.0 Na, 0.2 Ca, 0.05 Mg (mM)	0.28	2.8	40.81	4.37	1.13	6.07
pH	7.3	0.08 K, 2.0 Na, 0.2 Ca, 0.05 Mg (mM)	0.18	2.68	37.19	4.49	1.15	5.05
pH	7.6	0.08 K, 2.0 Na, 0.2 Ca, 0.05 Mg (mM)	0.11	3.16	29.94	4.26	1.02	5.69

In the pH test, dose-response curves overlapped in the low pH range, while they differed at high pH values (Fig. 1c–f). At pHs of 4.5 to 7.6, the values of EC_50_(Cu^2+^) varied by 4.5-fold and those of EC_50_(Co^2+^) varied by a factor of 4.3 (Table 1). However, there was a non-linear relationship between EC_50_{M^2+^}(M^2+^: Cu^2+^, Co^2+^) and H^+^ activities, which indicated that the effect of pH on EC_50_{M^2+^} cannot be explained by H^+^ competition. Early research indicated that the pH effect on metals may be related to the speciation of metals^13^. Thus, we determined the relationship between Cu or Co species and their toxicity using a previously described method^13^. We found that the contribution of CuCO_3_(aq) and CuOH^+^ to toxicity should be considered at high pH values. Similar results were found for Co. Furthermore, we determined stability constants (log*K*) for binding of Mg^2+^, Cu^2+^ and Co^2+^ of Cu-only and Co-only systems based on the BLM method for single metals (Table 2). For details, refer to De Schamphelaere^20^. In addition, the conditional binding constants of the inorganic metal complexes such as CuOH^+^ were also evaluated based on single toxicity data. The results were $\mathrm{log}\,{K}_{{{\rm{CuHCO}}}_{3}{\rm{BL}}}$ = 5.67, $\mathrm{log}\,{K}_{{{\rm{CuCO}}}_{3}{\rm{BL}}}$ = 5.44, log*K*_CuOHBL_ = 5.07 and $\mathrm{log}\,{K}_{{{\rm{CoHCO}}}_{3}{\rm{BL}}}$ = 5.81.

**Table 2:** Fitted parameters for the BLM derived from Cu-only and Co-only; and fitted parameters from the FIAM-TU and BLM-TU models derived from Cu–Co mixture.

Model	Log*K*_Mg_	Log*K*_MBL_	${\bf{log}}\,{K}_{{{\bf{CuHCO}}}_{3}{\bf{BL}}}$	log*K*_CuOHBL_	$\mathrm{log}\,{K}_{{{\bf{CuCO}}}_{3}{\bf{BL}}}$	${\bf{log}}\,{K}_{{{\bf{CoHCO}}}_{3}{\bf{BL}}}$	*x*_50_	*β*
Cu-only	2.93	5.87	5.67	5.07	5.44	‒	0.28	2.73
Co-only	3.84	4.72	‒	‒	‒	5.81	0.66	4.59
FIAM-TU	‒	‒	‒	‒	‒	‒	0.87	1.71
BLM-TU	‒	‒	‒	‒	‒	‒	1.45	4.04

### Combined toxicity of Cu–Co mixtures

**Figure 2:**
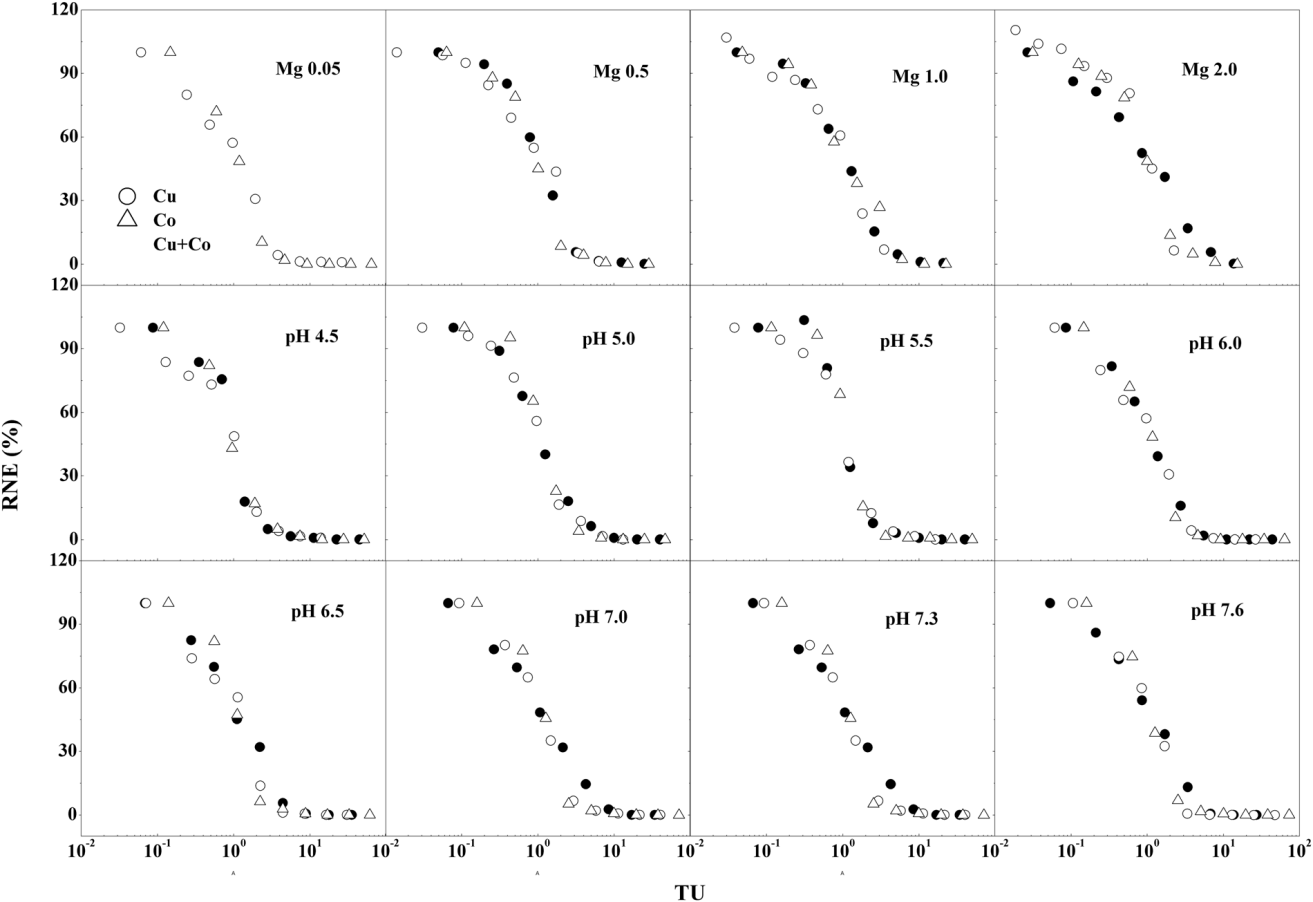
Response of wheat roots exposed to single Cu (•), Co (○) and their combinations (Δ) shown as the relationship between RNE and toxic unit values (TU_M_, calculated on the basis of free Cu^2+^ and Co^2+^ activity and Eqn. 1) under different Mg treatments (first row), at pH 4.5 to 6.0 (second row) and at pH 6.5 to 7.6 (third row). Each data point represents the RNE at the corresponding Mg and pH treatments.

The observed percentage of wheat root elongation (RNE%) of individual Cu and Co and Cu–Co mixtures expressed as the TU_M_ (calculated on the basis of free Cu^2+^ and Co^2+^ activity) for each Mg^2+^ treatment and pH values are shown in Fig. 2. The observed toxic effects of individual Cu, Co, and Cu–Co mixture showed no obvious deviations in any sets, which indicates that the observed toxicity of Cu–Co mixtures was the same for the individual Cu and Co systems. Then, the TU_M_ values for each Mg^2+^ treatment and pH values of Cu–Co mixtures were calculated based on Eqn. 1, and the TU_M_ at 50% RNE (TU_50_) were fitted based on Eqn. 5. The values of TU_50_ for all Cu–Co sets were close to 1 (0.88–1.15; Table 1). These results show that the Cu–Co mixture followed a trend of additive effects.

### Predicted Cu–Co mixture toxicity based on the BLM

**Figure 3:**
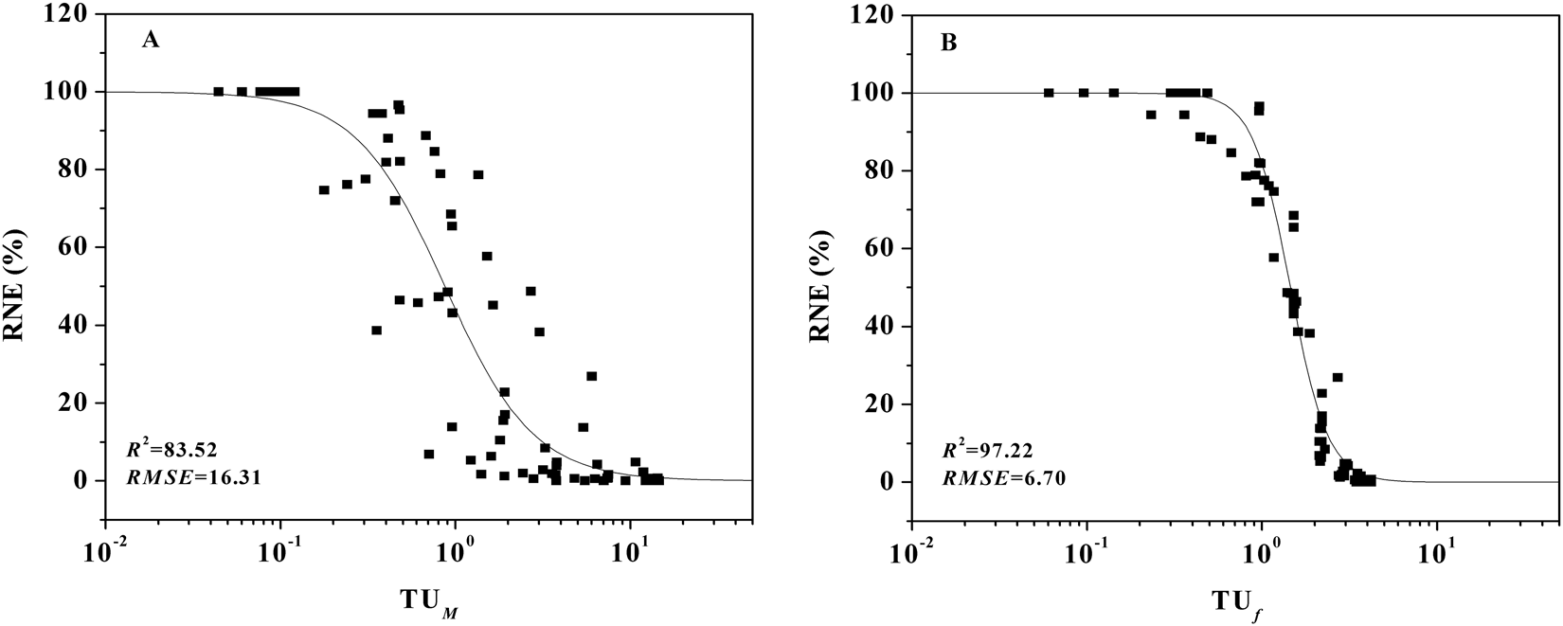
Dose–response curves for toxicity of Cu–Co combinations shown as the relationship between RNE and TU calculated by TU_M_ (A) and TU_*f*_ (B). *R* ^2^ is the determination coefficient of the models between the measured and predicted RNE. *RMSE* is the root mean square error of the models. The scatter points represent observed mixture toxic effects and the solid line is the predicted mixture toxic effects. In A, data were fitted using: RNE (%) = 100/{1 + [(M_Cu_/EC50_Cu_ + M_Co_/EC50_Co_)/${{\rm{TU}}}_{{\rm{M}}}^{{\rm{50}}}$]^*β*^_M_}. In B, the data were fitted using Eqn: RNE (%) = 100/{1 + [(*f*_Cu_ /${f}_{{\rm{CuBL}}}^{50 \% }$ + *f*_Co_/${f}_{{\rm{CoBL}}}^{50 \% }$)/${{\rm{TU}}}_{f}^{{\rm{50}}}$]^*β*^_M_}.

The TU_50_ being equal to 1 for all mixed sets revealed that there was no interaction between Cu^2+^ and Co^2+^. Therefore, TU_*f*_ and TU_M_ were proposed for use in predicting Cu–Co toxicity according to Eqn. 5 and in developing the BLM-based and FIAM-based TU models in the present study. For every treatment (12 bioassays × 8 concentrations of Cu–Co mixture) the TU_*f*_ or TU_M_ value was calculated using Eqn. 4 for varying *f* or M (free Cu^2+^ and Co^2+^ activity), respectively; where *f* can be calculated based on the activities of free Cu^2+^, Co^2+^, Mg^2+^ and inorganic metal complexes in the Cu–Co mixture and Log*K* values derived from the individual Cu and Co systems (Eqn. 3). Thus, the combined toxicity of Cu–Co mixtures could be expressed using the TU_*f*_ or TU_M_ values (Eqn. 5). The predicted dose–response curves and fitted parameters of all sets with the BLM-based and FIAM-based TU approaches are shown in Fig. 3 and Table 2. The root mean square error (*RMSE*) was calculated for each prediction (Fig. 3). The BLM-based TU approach performed better for predicting root elongation than the FIAM-based TU model based on *RMSE* and *R* ^2^ values. Specifically, the *RMSE* value between the observed and predicted RNE of the BLM (6.70) was much lower than that of the FIAM (16.31). This was likely due to the inclusion of the competition between Cu or Co and Mg^2+^ for binding sites on wheat roots and consideration of the effects of CuHCO_3_^+^, CuCO_3_(aq), CuOH^+^ and CoHCO_3_^+^, under various pH levels, on the estimation of the *f* values. Therefore, the proposed BLM-based TU was a better method to predict the toxicity of Cu–Co mixtures for wheat.

## Discussion

The observed EC_50_ for free Cu^2+^ or Co^2+^ activity increased up to 2.5-fold and 10-fold with increasing Mg^2+^ concentrations, respectively. These findings indicate that Mg^2+^ had a protective effect against the toxicity of Cu or Co, which is similar to previous studies^[13,17,19]^. For instance, Luo *et al*. reported that Mg^2+^ can alleviate Cu toxicity for wheat in nutrient solution, and calculated that the EC_50_ for free Cu^2+^ increased by up to 3.7-fold. Wang *et al*. carried out similar root elongation tests on barley using a growth solution with a series of Mg^2+^ (0.05–2 mM) concentrations and found an increase in EC_50_ of free Cu^2+^ activity by a factor of 3. Lock *et al*.^19^. reported that the increase of Mg^2+^ concentrations could alleviate Co toxicity to barley, and calculated a 15.6-fold increase in EC_50_ of free Co^2+^ activity in solution. The effect of Mg^2+^ on Cu^2+^ and Co^2+^ toxicity may be due to similar ionic radii of Mg^2+^, Co^2+^ and Cu^2+^, or due to competition for transporters^21^.

The present study revealed that the EC_50_ of Cu or Co decreased by up to 4.1-fold with increasing pH values; however, there was no obvious linear relationship between H^+^ and EC_50_. These findings differ from those for the BLM with H^+^, which can compete with free metal activity ions at BL. Therefore, it is unjustified to incorporate H^+^ competition into the BLM. The present study indicated that the effects of pH on the toxicity of metals may be due to the contribution of inorganic metal complexes to toxicity. For instance, De Schamphelaere and Janssen suggested that CuOH^+^ contributed to Cu toxicity in *Daphnia magna* ^22^. For terrestrial plants, Wang *et al*.^13^ indicated that when incorporating inorganic species of Cu^2+^ into the BLM, the regression coefficient (*R* ^2^) between the measured and predicted EC_50_{Cu^2+^} values was as high as 0.97. These findings suggested that some species of inorganic metal complexes were toxic and should be considered in BLMs with high pH values. The present study indicates that incorporating inorganic metal complexes when assessing the joint toxicity of Cu–Co improves the predictive capacity of the BLM.

The binding constants derived in the present study for wheat can be compared with those reported for Cu-BLM^13^ and Co-BLM^19^. The values of log*K*_CuBL_ (5.87), $\mathrm{log}\,{K}_{{{\rm{CuHCO}}}_{3}{\rm{BL}}}$ (5.67), $\mathrm{log}\,{K}_{{{\rm{CuCO}}}_{3}{\rm{BL}}}$ (5.44), log*K*_CuOHBL_ (5.07) and log*K*_MgBL_ (2.93) in the present study were closer to the results reported by Wang *et al*.^13^. In addition, the values of log*K*_CoBL_ (4.72) were slightly lower than those of (log*K*_CoBL_ = 5.13) reported by Lock *et al*.^19^, whereas they were very similar to those (log*K*_CoBL_ = 4.70) published by Garnham *et al*.^23^. The log*K*_MgBL_ (3.84) of Co was similar to that reported by Lock *et al*. (log*K*_MgBL_ = 3.95)^19^. Different exposure duration, endpoint, target tissue or BL, or mechanisms of Cu or Co uptake resulted in differences in binding constants^16^.

The present study showed that CoHCO_3_^+^ had 11-fold higher binding affinity than Co^2+^, similar to the results of Deleebeeck *et al*. and Wang *et al*., who reported that the affinities of inorganic complexes of metals for the BL were higher relative to the free metal ion^[13,24,25]^. However, it is mechanistically very unlikely that BL constants for complexes of metals are very close to or higher than for the free metal ion. Therefore, the binding of inorganic metal complexes with BL needs further investigation and direct evidence.

It is widely believed that most metal combinations act additively. For example, Ownby and Newman *et al*. reported that inhibition of bioluminescence in *Microtox* assays was approximately additive in Cd–Cu mixtures^26^. Marra *et al*. found that interactions of Cu–Co mixtures were additive for rainbow trout and duckweed^27^. The present study indicated that the joint toxicity of Cu and Co was additive for wheat. These results are similar to those reported by Ownby and Newman *et al*. and Marra *et al*.^26, 27^. However, some researchers pointed out that the joint toxicity of mixtures of metals may exhibit synergistic or antagonistic effects, rather than simply being additive. Versieren *et al*.^10^ showed significant (*p* < 0.05) antagonistic interactions between Cu–Zn and lettuce at low Ca^2+^ concentrations. Ince *et al*. described Cu–Co interactions that were synergistic at most test levels for *Vibrio fisheri* but that had additive effects under some individual conditions^28^. Thus, the difference in interactions between metal ions may be due to exposure time and test species^29^. The present study indicated that the BLM-based TU approach, which accounted for metal speciation and the integrated competition effects of the major cations, was more accurate at predicting the combined toxicity of Cu–Co mixtures than the FIAM-based TU model. Future studies should investigate the biological actions of metals in plant cell compartments following exposure to mixtures of metals to provide better insight into the mechanisms.

## Conclusion

In summary, a BLM-based TU was developed to predict the combined toxicity of Cu–Co mixtures at different Mg^2+^ activities and at various pH levels for wheat in nutrient solutions. Using the estimated constants based on the individual Cu and Co toxicity data, the BLM-based TU approach more accurately predicted the joint Cu–Co toxicity than the FIAM-based TU approach. Further research is required to investigate the toxicity of metal mixtures in a wide range of natural or field soils before BLMs can be used for risk assessments of metal co-contaminated soil in the field.

## Materials and Methods

### Experimental design

Wheat root elongation tests were used to evaluate the toxicity of individual Cu, Co and Cu–Co mixtures (molar ratio of Cu:Co = 1:100) in a simplified culture solution. Two sets of experiments were conducted: a Mg-set and a pH-set (Table 1). Each set included a series of media with different Mg^2+^ concentrations and varied pH values. The concentrations of background electrolytes were selected based on previously reported data^13^ (Table 1). For single-metal system tests, seven Cu^2+^ (0.2–12.8 μM), seven Co^2+^ (20–1280 μM), seven Cu^2+^–Co^2+^ concentrations and one control solution (without Cu^2+^ or Co^2+^) were prepared. All treatments were performed in triplicate.

### Preparation of test media

All experiments used chemicals of analytical reagent grade or higher, and deionized water was used throughout. Tested solutions were prepared by adding different volumes of stock solutions of CaCl_2_, MgSO_4_, NaCl and KCl into deionized water. Additionally, the buffer solutions used were 1 mM 2-[N-morpholino] ethane sulfonic acid for pH < 7.0 treatments and 3.6 mM 3-[N-morpholino] propane sulfonic acid for pH ≥ 7.0^30^. Moreover, dilute NaOH or HCl were used to adjust pH to the desired level for each pH-set, and pH was controlled at 6.0 for the Mg-set. The pH values of solutions were measured before and after the bioassay. The different chemical characteristics of the test media are shown in Table 1.

### Toxicity tests

Wheat root elongation tests were performed following ISO Guideline 11269-1(1993). The test seeds of wheat (*T*. *aestivum* L. cv. Zongmai 335) were purchased from the Chinese Academy of Agricultural Sciences (Beijing, China). Wheat seeds were sterilized for 30 min in 30% H_2_O_2_, then thoroughly rinsed with deionized water and germinated at 25 ± 1 °C in darkness for 48 h on moistened filter-paper^22^. After the radicle emerged (about 1 cm in length), six seeds were transferred to a nylon net fixed on the surface of the plastic culture pots containing 350 mL of the solution. The air temperature of the growth chamber was maintained at 20 ± 1 °C for 48 h in darkness and the culture pots were randomly placed in the growth chamber. The lengths of the longest roots on each seedling were measured after 2 d, and the mean value of the three replications for each test was used for data analysis. The relative net elongation (RNE) was calculated and expressed as the percentage relative to the control (Eqn 2):

2 $$RNE \% =\frac{(R{E}_{t}-R{E}_{0})}{(R{E}_{c}-R{E}_{0})}$$

where *RE*_*t*_ is the root length in the metal treatment, *RE*_0_ is the original length and *RE*_*c*_ is the root length in the control.

### Chemical analyses

The concentrations of Cu, Co and Mg in test solutions were measured by inductively coupled plasma optical emission spectrometry (720-ES, Varian, Palo Alto, CA, USA). The solution pH was determined using a pH meter (Delta 320; Mettler, Zurich, Switzerland).

### Prediction of Cu and Co speciation in solutions

WHAM 6.0 (Windermere Humic Aqueous Model) was used to calculate the speciation of Cu and Co^31^. The pH and measured total concentrations of Cu^2+^, Co^2+^, K^+^, Na^+^, Ca^2+^, Mg^2+^, Cl^−^ and SO_4_^2−^ were inputted into WHAM. For details, refer to Lofts *et al*.^31^. The calculated proportions of free Cu^2+^ and Co^2+^ activity (% of total Cu or Co) accounted for 11.7–78.6% and 61.5–78.6% with pH increasing from 4.5 to 7.6, respectively. The calculated proportions of CuHCO_3_^+^, CuCO_3_, CuOH^+^ and CoHCO_3_^+^ were 0.25–46.6, 0.00–25.0, 0.05–9.54 and 0.01–9.74%, respectively.

The Data Processing System 9.0 (DPS9.0), developed by Tang and Feng^32^, was used to estimate the parameter values of the BLM-based and FIAM-based TU models.

### Mathematical description of the BLM and derivation of parameters

The BLM methodology is based on the assumption that stability constants remain the same under various physico-chemical conditions^20^. The following is a short mathematical description of the BLM along with the equations required to understand the calculations. Based on the BLM assumption, the fraction (*f*) of the total number of BL sites occupied by Cu^2+^ or Co^2+^ is given by the following equation when the competing cations and toxicity of inorganic metal complexes are considered:

3 $$f=\frac{{K}_{{\rm{MBL}}}\{{{\rm{M}}}^{2+}\}+\sum {K}_{{\rm{IMBL}}}\{\mathrm{IM}\}}{{K}_{{\rm{MBL}}}\{{{\rm{M}}}^{2+}\}+\sum {K}_{{\rm{IMBL}}}\{\mathrm{IM}\}+\sum {K}_{{\rm{XBL}}}\{{X}^{n+}\}}$$

where, *K*_MBL_, *K*_IMBL_ and *K*_XBL_ are constants for the binding of free Cu^2+^ or Co^2+^ (M), inorganic Cu^2+^ or Co^2+^ complexes (IM) and cation X (e.g., Mg ^2+^) to the BL sites, respectively, and brackets {} indicate ion activity, such as {X ^n+^}, which is the activity of X^n+^ (M). For mixtures of metals, substituting *f* from Eqn. 3 into Eqn. 1, transforms Eqn. 1:

4 $${{\rm{TU}}}_{f}=\sum {\rm{TU}}=\frac{{f}_{{\rm{Cu}}}}{{f}_{{\rm{CuBL}}}^{50 \% }}+\frac{{f}_{{\rm{Co}}}}{{f}_{{\rm{CoBL}}}^{50 \% }}$$

where ${f}_{{\rm{CuBL}}}^{50 \% }$ and ${f}_{{\rm{CoBL}}}^{50 \% }$ are the fraction of BL sites occupied by Cu or Co, respectively, at 50% RNE under single metal exposure. The TU_*f*_ values can be used to establish the BLM-based TU approach. When *f* is replaced with free ion activity of M (Cu^2+^ or Co^2+^), the TU_M_ values can be calculated and used to establish the FIAM-based TU model.

The wheat root elongation was correlated with TU_*f*_ or TU_M_ and was assumed to follow a log-logistic dose–response relationship according to Thakali *et al*.^33^

5 $$\mathrm{RNE},\,R( \% )=\frac{100}{1+{(\frac{x}{{x}_{50}})}^{{\beta }_{{\rm{M}}}}}$$

where *β*_*M*_ is a fitting parameter determining the slope of the dose–response curve; *x* is the value of the toxicity index, i.e. TU_*f*_ or TU_M_; and *x*_50_ is the value of TU at 50% RNE. Eqn. 5 was applied to predict the mixture toxicity effects and compare the BLM-TU and FLAM-TU approaches.

## Electronic supplementary material

Supporting Information
