# Supplementary material for: Modeling acute toxicity of metal mixtures to wheat (Triticum aestivum L.) using the biotic ligand model-based toxic units method
Source: Sci Rep. 2017 Aug 25;7:9443. doi: 10.1038/s41598-017-09940-5 (PMC5573337; doi:10.1038/s41598-017-09940-5)
Supplement: Supplementary file 1 — Supporting Information [file 41598_2017_9940_MOESM1_ESM.pdf]

Modeling acute toxicity of metal mixtures to wheat (*Triticum aestivum* L.) using the biotic ligand model-based toxic units method

Mingyan Wu[1], Xuedong Wang[1*], Zhiguo Jia[2], Karel De Schamphelaere[3], Dongxue Ji[1], Xiaoxiu Li[1], Xiaolin Chen[1]

[1] The Key Lab of Resource Environment and GIS, College of Resource Environment and Tourism, Capital Normal University, Beijing 100048, P.R. China

[2] Department of Horticulture, Hebei North University, Zhangjiakou 075000, P.R. China

[3] Laboratory of Environmental Toxicology and Aquatic Ecology, Environmental Toxicology unit (GhEnToxLab), Ghent University, Gent B-9000, Belgium

AUTHOR INFORMATION

***Corresponding Author**

105 North road of Xisanhuan, Beijing 100048, China

E-mail: xdwang@cnu.edu.cn

Supporting Information

Table S1 Added Cu and Co concentrations (μM) in the nutrient solutions and calculated corresponding elongation (RE) of wheat (*T. aestivum* L.) for different Mg^{2+} activities and pH treatments.

Exp.1	Single Cu		Single Co		Cu+Co		
	Cu (μM)	RE %	Co (μM)	RE %	Cu (μM)	Co (μM)	RE %
0.05 mM Mg	0	100.0	5	100.0	0.05	5	100.0
	0.2	81.9	20	80.0	0.2	20	72.0
	0.4	65.2	40	65.9	0.4	40	48.5
	0.8	39.4	80	57.2	0.8	80	10.5
	1.6	16.0	160	30.8	1.6	160	1.9
	3.2	1.2	320	4.3	3.2	320	0.1
	6.4	1.0	640	1.2	6.4	640	0.1
	12.8	0.9	1280	1.0	12.8	1280	0.1
0.5 mM Mg	0	100.0	5	100.0	0.05	5	100.0
	0.2	94.4	20	98.6	0.2	20	88.0
	0.4	85.2	40	95.1	0.4	40	78.9
	0.8	59.9	80	84.5	0.8	80	45.1
	1.6	32.4	160	69.0	1.6	160	8.5
	3.2	5.6	320	54.9	3.2	320	4.2
	6.4	1.5	640	43.7	6.4	640	0.7
	12.8	0.8	1280	5.3	12.8	1280	0.1
1.0 mM Mg	0	100.0	5	100.0	0.05	5	100.0
	0.2	94.6	20	106.9	0.2	20	94.4
	0.4	85.4	40	96.9	0.4	40	84.6
	0.8	63.9	80	88.5	0.8	80	57.7
	1.6	43.9	160	86.9	1.6	160	38.3
	3.2	15.4	320	73.1	3.2	320	26.9
	6.4	4.6	640	60.8	6.4	640	2.3
	12.8	1.0	1280	23.9	12.8	1280	0.1
2.0 mM Mg	0	100.0	5	100.0	0.05	5	100.0
	0.2	86.3	20	110.5	0.2	20	94.4
	0.4	81.0	40	104.0	0.4	40	88.7
	0.8	69.4	80	101.6	0.8	80	78.6
	1.6	52.4	160	93.6	1.6	160	48.7
	3.2	41.1	320	87.9	3.2	320	13.7
	6.4	16.9	640	80.7	6.4	640	4.8
	12.8	5.7	1280	45.2	12.8	1280	0.8

pH 4.5	0	100.0	5	100.0	0.05	5	100.0
	0.2	83.7	20	83.7	0.2	20	82.1
	0.4	75.6	40	77.2	0.4	40	43.1
	0.8	17.9	80	73.2	0.8	80	17.1
	1.6	4.9	160	48.8	1.6	160	4.9
	3.2	1.6	320	13.0	3.2	320	1.6
	6.4	0.8	640	4.1	6.4	640	0.0
	12.8	0.0	1280	1.6	12.8	1280	0.0
pH 5.0	0	100.	5	100.0	0.05	5	100.0
	0.2	89.1	20	96.1	0.2	20	95.4
	0.4	67.8	40	91.4	0.4	40	65.4
	0.8	40.2	80	76.4	0.8	80	22.9
	1.6	18.1	160	56.0	1.6	160	3.9
	3.2	6.3	320	16.6	3.2	320	0.8
	6.4	0.8	640	8.7	6.4	640	0.0
	12.8	0.0	1280	1.6	12.8	1280	0.0
pH 5.5	0	100.0	5	100.0	0.05	5	100.00
	0.2	103.6	20	94.2	0.2	20	96.57
	0.4	81.0	40	88.0	0.4	40	68.54
	0.8	34.3	80	77.9	0.8	80	15.58
	1.6	7.8	160	36.6	1.6	160	1.56
	3.2	3.1	320	12.5	3.2	320	0.78
	6.4	0.8	640	3.9	6.4	640	0.78
	12.8	0.0	1280	1.6	12.8	1280	0.0
pH 6.0	0	100.0	5	100.0	0.05	5	100.0
	0.2	81.8	20	80.0	0.2	20	72.0
	0.4	65.2	40	65.8	0.4	40	48.4
	0.8	39.4	80	57.2	0.8	80	10.5
	1.6	16.0	160	30.8	1.6	160	1.9
	3.2	1.9	320	4.3	3.2	320	0.0
	6.4	0.0	640	0.6	6.4	640	0.0
	12.8	0.0	1280	0.0	12.8	1280	0.0
pH 6.5	0	100.0	5	100.0	0.05	5	100.0
	0.2	82.5	20	80.0	0.2	20	81.9
	0.4	69.9	40	65.8	0.4	40	47.3
	0.8	45.3	80	57.2	0.8	80	6.3
	1.6	32.1	160	30.8	1.6	160	2.0
	3.2	5.7	320	4.3	3.2	320	0.6
	6.4	0.6	640	0.6	6.4	640	0.0
	12.8	0.0	1280	0.0	12.8	1280	0.0
pH 7.0	0	100.0	5	100.0	0.05	5	100.0
	0.2	78.2	20	80.2	0.2	20	77.5
	0.4	69.6	40	64.9	0.4	40	45.7
	0.8	48.4	80	35.1	0.8	80	5.3

	1.6	31.8	160	6.6	1.6	160	2.0
	3.2	14.6	320	2.0	3.2	320	0.7
	6.4	2.7	640	0.7	6.4	640	0.0
	12.8	0.0	1280	0.0	12.8	1280	0.0
pH 7.3	0	100.0	5	100.0	0.05	5	100.0
	0.2	76.7	20	79.7	0.2	20	76.1
	0.4	68.2	40	64.0	0.4	40	46.
	0.8	50.1	80	32.00	0.8	80	13.9
	1.6	39.9	160	6.0	1.6	160	1.2
	3.2	10.9	320	0.6	3.2	320	0.6
	6.4	3.6	640	0.0	6.4	640	0.6
	12.8	0.0	1280	0.0	12.8	1280	0.0
pH 7.6	0	100.0	5	100.0	0.05	5	100.0
	0.2	86.0	20	74.6	0.2	20	74.7
	0.4	73.5	40	59.8	0.4	40	38.7
	0.8	54.1	80	32.5	0.8	80	6.8
	1.6	38.2	160	0.6	1.6	160	1.7
	3.2	13.1	320	0.1	3.2	320	0.6
	6.4	0.6	640	0.0	6.4	640	0.0
	12.8	0.0	1280	0.0	12.8	1280	0.0

Mathematical description of the BLM and derivation of parameters

Below, a short mathematical description of the BLM is given. A more detailed description of the method can be found in De Schamphelaere and Janssen[1]. Using this approach, the development of the BLMs to predict Cu toxicity to wheat and earthworm[2,3] as well as Co and Ni toxicity to barley[4,5] were realized.

Based on the BLM assumption, when the competing cation $Mg^{2+}$was considered, the fraction (*f*) of the total biotic ligand sites bound by Cu^{2+} or Co^{2+} is given by the following equation[1]:

$$f_{MBL} = \frac{K_{MBL}\{\mathrm{M}^{2+}\}}{1 + K_{MBL}\{\mathrm{M}^{2+}\} + \sum K_{XBL}\{\mathrm{X}^{n+}\}} \quad (1)$$

where K_{MBL} and K_{XBL} are conditional binding constants for the binding of Cu^{2+} or Co^{2+} and cation X (e.g., Mg^{2+}) to the BL sites (M), respectively, and brackets { }

indicates the ion activity, e.g. $\{X^{n+}\}$ presents the activity of X^{n+} (M). {XBL} is the concentration of the specific cation–BL complex (M).

According to the methodology described by Pagenkopf[6] and De Schamphelaere and Janssen[1], when inhibition of barley root elongation is up to 50% of the control, Eq. (1) becomes:

$$\mathrm{EC}_{50}(\mathrm{M}) = \frac{f_{MBL}^{50}}{(1 \quad f_{MBL}^{50})K_{MBL}}\left(1 + \sum K_{XBL}[\mathrm{X}]\right) \qquad (2)$$

Where EC_{50} (M) is the free Cu^{2+} or Co^{2+}that results in 50% RNE (50% of wheat root elongation with respect to the control) and $f_{MBL}^{50\%}$ is the fraction of the BLs that results in 50% RNE when occupied by Cu or Co. Eq. (2) shows that the linear relationships should be observed between $EC_{50}\{M^{2+}\}$ and the activity of one cation when other cation activities are kept constant, if the BLM concept is correct. The slopes and the intercepts of these linear relationships can then be used to derive the conditional binding constants of the competing cations according to Eq. (2) and Eq. (3). For example, when Mg^{2+} is varied, the following R_{Mg} ratio between the slope and the intercept can be derived from the linear regression analysis between the EC50(Cu) and the Mg^{2+} activity and Eq. (2):

$$\mathrm{R}_{\mathrm{Mg}} = \frac{\mathrm{Slope}_{\mathrm{Mg}}}{\mathrm{Intercept}_{\mathrm{Mg}}} = \frac{K_{MgBL}}{(1 + \sum K_{XBL}[\mathrm{X}]} \qquad (3)$$

Consequently K_{MBL} and $f_{MBL}^{50\%}$ can be calculated based on the optimization of the logit-transformed effect versus f_{MBL} for varying K_{MBL}.

Reference

1. De Schamphelaere, K.A.C., Janssen, C.R. A biotic ligand model predicting acute copper toxicity for Daphnia magna: The effects of calcium, magnesium, sodium, potassium, and pH. Environ. Sci. Technol. 36, 48–54 (2002).

2. Luo, X.S., Li, L.Z., Zhou, D.M. Effect of cations on copper toxicity to wheat root: Implications for the biotic ligand model. Chemosphere. 73, 401– 406 (2008).

3. Steenbergen, N.T., Iaccino, F., De Winkel. M., Reijnders, L., Peijnenburg, W.J. Development of a biotic ligand model and a regression model predicting acute copper toxicity to the earthworm Aporrectodea caliginosa. Environ Sci Technol 39: 5694–5702 (2005).

4. Lock, K., De Schamphelaere, K.A.C., Because, S., Criel, P., Van Eeckhout, H., Janssen, C.R. Development and validation of a terrestrial biotic ligand model predicting the effect of cobalt on root growth of barley (*Hordeum vulgare*). Environ. Pollut. 147, 626–633 (2007a).

5. Lock, K., Van Eeckhout, H., De Schamphelaere, K.A.C., Criel, P., Janssen, C.R. Development of a biotic ligand model (BLM) predicting nickel toxicity to barley (*Hordeum vulgare*). Chemosphere. 66, 1346–1352 (2007b).

6. Pagenkopf, G.K. Gill surface interaction model for trace metal toxicity to fishes: Role of complexation, pH and water hardness. Environ. Sci. Technol. 17, 342–347 (1983).
